# Artificial Intelligence Methods for Constructing Wine Barrels with a Controlled Oxygen Transmission Rate

**DOI:** 10.3390/molecules25143312

**Published:** 2020-07-21

**Authors:** Víctor Martínez-Martínez, Ignacio Nevares, Maria del Alamo-Sanza

**Affiliations:** 1 Department of Agricultural and Forestry Engineering, UVaMOX-Universidad de Valladolid, 34001 Palencia, Spain; victor.martinez.martinez@uva.es; 2 Department of Analytical Chemistry, UVaMOX-Universidad de Valladolid, 34001 Palencia, Spain

**Keywords:** genetic algorithm, active packaging, oxygen transmission rate, cooperage, process optimization

## Abstract

Oxygen is an important factor in the wine aging process, and the oxygen transmission rate (OTR) is the parameter of the wood that reflects its oxygen permeation. OTR has not been considered in the cooperage industry yet; however, recent studies proposed a nondestructive method for estimating the OTR of barrel staves, but an efficient method to combine these staves to build barrels with a desired OTR is needed to implement it in the industry. This article proposes artificial intelligence methods for selecting staves for the construction of barrel heads or bodies with a desired target OTR. Genetic algorithms were used to implement these methods in consideration of the known OTR of the staves and the geometry of the wine barrels. The proposed methods were evaluated in several scenarios: homogenizing the OTR of the actual constructed barrels, constructing low-OTR and high-OTR barrels based on a preclassification of the staves and implementing the proposed method in real cooperage conditions. The results of these experiments suggest the suitability of the proposed methods for their implementation in a cooperage in order to build controlled OTR barrels.

## 1. Introduction

Wine has been traditionally aged in barrels in many wine-growing regions around the world. This procedure improves the chemical and sensorial characteristics of the aged wines, thereby improving the quality of the resulting wine. During the aging process, the wine properties evolve due to the interaction between the wine compounds and the compounds released from the wood, and the oxygen that enters through the barrel is a key factor in wine evolution.

The barrel making process is a traditional procedure in which several staves are combined to build a barrel. This process has been traditionally performed by considering the shape of the barrel, its capacity, and the wood that is used, which are design parameters that have changed throughout history and vary across wine-producing regions [1]. Moreover, barrel shape and size have been mathematically modeled in the literature to relate the barrel volume [2,3] and its surface area [4] with the dimensions of the barrel staves. The problem of selecting the staves for the construction of a barrel can be regarded as a mathematical multiobjective optimization problem that can be divided into two sub-problems. The first sub-problem is selecting a set of staves with a total width equal to the width of the barrel body, with at least one stave having sufficient width for the barrel bung. The second sub-problem is selecting two sets of staves that fulfill the size requirements of the barrel heads. Currently, this procedure is performed by an expert worker (Master Cooper), who chooses the staves for the construction of each barrel without any technological aid. Moreover, only the geometrical characteristics of the staves are considered in the selection of the staves; other wood properties, such as the oxygen transmission rate (OTR), are not considered.

Several authors have analyzed the relations between the wood and the gas permeation under a pressure difference [5,6,7,8,9,10,11], although very few studies refer to the transverse permeability of wood to gases as being the driving force of the oxygen concentration or consider the influence that the moisture level of the wood exerts on this gaseous flow [12,13,14,15,16,17], which is the real scenario in a barrel oak stave. The work of Nevares et al. [18] showed that despite the strong role of the anatomical characteristics of *Quercus petraea* wood, these characteristics are not sufficient for fully explaining the capacity for transferring oxygen or the variability in this transmission rate (OTR) via the simple correlations that have been studied. Nevertheless, Martínez-Martínez et al. [19] proposed a method for estimating the OTR of oak wood samples via nondestructive methods that are based on artificial neural networks in consideration of not just one but many of the anatomical features of the oak wood. Moreover, this method could be implemented in a cooperage because the required equipment and processing time enable its use in a production line. Moreover, the work of Prat-García et al. [4] demonstrated that there are significant differences in the permeation to oxygen among the low-OTR and high-OTR barrels that are constructed by applying this method. Therefore, to implement these previously obtained scientific results on a cooperage production line, it will be necessary for an automatic staves selection method to construct barrels with a desired OTR taking into consideration the OTR of the staves.

Several methods are available for automating the resolution of multiobjective optimization problems. The first group of methods are brute-force (BF) based methods, which evaluate all the possible solutions and choose the best solution. BF-based methods find the best solution for the problem, but they have higher computation and time requirements compared with other methods, which render them unsuitable for use in complex problems. For this reason, these methods have been used in many fields, but always as a part of a more complex method or with modifications that improve their performance when the complexity of the problem increases [20]. The second group of methods are the Monte Carlo (MC) approaches [21], which evaluate solution candidates that have been randomly chosen from the solution set and selects the best solution among all evaluated candidates. Compared with BF-based methods, MC methods can find satisfactory solutions in less processing time. Nevertheless, when the complexity of the problem increases, more complex methods are needed to find satisfactory solutions to the problem in a reasonable processing time. Artificial intelligence (AI)-based methods are one of the best options for solving multiobjective nonlinear optimization problems, improving the performance of BF- and MC-based methods. Examples of AI-based methods include ant colony optimization [22], artificial bee colony [23], harmony search [24], particle swarm optimization (PSO) [25,26] and genetic algorithms (GAs) [27,28]. GAs are some of the most frequently used AI-based methods due to their flexibility and performance in modeling all types of processes. Related to the work that is presented in this article, GA-based methods have been applied in the development of several industrial applications to increase the productivity [29]. Furthermore, GA-based methods have been used in industrial application for quality control and defect detection [30,31], to solve packaging problems [32,33] and to forecast production [34,35,36] and energy demand [37]. Moreover, GA-based algorithms have been used in the wine field: Burratti et al. combined electronic nose, electronic tongue and spectrophotometric measurement data for the prediction of sensorial descriptors [38]; Beltrán et al. used GAs to extract features from high-performance liquid chromatograph data for the classification of Chilean wines [39]; Cao et al. predicted pH and soluble solids content and discriminated the variety of grapes with a non-destructive method based on visible and near-infrared (Vis-NIR) spectroscopy [40]; Corcoran et al. used GAs to reduce the number of parameters that were obtained from multisensor arrays of sensors for the classification of wine samples [41]; and Kuo and Lin combined a GA and a PSO algorithm for clustering [42].

In this paper, GA-based methods for selecting the staves for the construction of a barrel are proposed. These methods consider not only the geometry of the staves, but also their OTR in the construction of barrels with a desired global OTR. The performance of the proposed method was analyzed with the data of 3064 oak wood staves, and it was compared with the performance of the method that is currently used in several cooperages and a MC-based approach to evaluate the improvement of the proposed method.

## 2. Materials and Methods

### 2.1. Oak Wood Samples

For the experiment, 3064 French oak (*Q. petraea*) fresh staves, which were provided by INTONA S.L. cooperage (Navarra, Spain) and were selected from among the oak samples that they use to construct regular barrels, were used to characterize the fresh stave population of this cooperage. The selection of these samples was conducted to obtain a representative sample of the wood that is used by this cooperage, and staves were selected from different batches and with various characteristics. These staves, which were widely analyzed in the work of Prat-Garcia et al. [4], were divided into 1836 fresh staves, with a length of approximately 96 cm, for the barrel body, and 1228, with a length of between 42 and 73 cm, for the barrel head, and a “grain” (width of the annual growth ring) value of between 1.88 and 4.94 mm. The OTR value of all the samples, which were measured by employing the ANN-based method of Martínez-Martínez et al. [19], and the widths of the body staves or the widths and lengths of the head staves were used as the dataset for the method evaluation that is presented in this article. A wider analysis of these samples can be found in the article of Prat-Garcia et al. [4].

In the real cooperage simulation experiment, where more samples than those available were necessary, the cumulative distribution function (CDF) of the features was used to generate the samples that were used in this experiment. Thus, the length, width and OTR data of the head staves and the length and OTR data of the body staves were regarded as representative of the cooperage stave population. Therefore, they were used to calculate their five associated CDFs. Then, three and two random vectors were initialized for the heads and bodies, respectively, with as many elements for each vector as head samples and body samples as were needed in each case. These random vectors were obtained from a continuous uniform distribution with values between zero and one. Finally, the CDFs of each feature were used to calculate the feature values from the random value vectors by using a linear interpolation to estimate the values for which the CDFs were not defined.

### 2.2. Barrel Construction Process

The barrels that are used to age beverages have three parts: the heads, which are the two circle parts; the body, which is the larger part that links the two heads; and the bung, which enables the barrel to be filled or emptied. Each cooperage company has its own procedure for constructing barrels with various shapes, volumes and properties. Nevertheless, the barrel construction process can be divided into three subprocesses: the barrel head construction process, the barrel body construction process and the barrel head and body assembly process. In the next paragraphs, the process that is used in this article will be explained.

A set of staves are needed for the construction of a circle of 597 mm in diameter in the head’s construction process. Thus, the length and width of the staves should be sufficient for constructing this circle. Another structural requirement is an odd number of staves so that there is one stave in the middle of the head. It makes that the number of staves employed to build a barrel head is, typically, 7, 9 or 11 staves. The order of the staves is relevant in the barrel construction because the length of the central stave of the head is larger than those of the staves of the extreme positions of the head.

There are two main requirements in the body construction process: at least one stave that is wider than 10 cm is needed for the placement of the barrel bung hole, and the total width of the staves must be 218 cm in a 225-L *Bordelaise barrel*. The number of staves needed to build a barrel body is around 30, but it can vary depending on the width of the staves employed. In the body construction process, in contrast to the head’s construction process, the position of the staves is not relevant. Some cooperages distribute the body staves by interleaving narrow and wide staves; however, this restriction was not considered in the barrel body GA method because the stave distribution does not affect the final OTR, as will be explained below. Thus, the cooper could choose from a set of staves to build a body barrel in which narrow and wide staves are interleaved, distributed randomly, or arranged using another criterion.

Finally, the barrel head and body assembly process consist of the assembly of two heads and a body to build the barrel, and there are no structural requirements regarding the parts to be assembled in this process.

## 3. Calculation

### 3.1. Head and Body OTR Calculation

The objective barrel OTR can be estimated from the staves OTR in consideration of the geometry of the barrel. Thus, the barrel OTR can be calculated as the sum of the estimated OTR for each stave and weighted by the ratio between each stave area and the total barrel area.

Two assumptions regarding the barrel characteristics were made in calculating the area ratio of each stave. The first assumption is that the barrel body area is 1.50 m^2^, and the second assumption is that each head barrel area is 0.25 m^2^. Thus, the body constitutes 74.77% of the total barrel area, and each head area constitutes 12.61% of the total barrel area. The method that is used to calculate this ratio differs according to whether the stave belongs to the body or to the heads. The OTR that is estimated via this method is only the OTR of the wood staves in the barrel, which is the main pathway (up to 75% of the overall OTR) of oxygen entry in French oak barrels [14]. To calculate the OTR of a barrel, the oxygen that flows between the staves must also be considered [13,43,44].

Considering these assumptions, the ratio for the barrel body and head staves can be calculated with the expressions presented in Equations (1) and (2) according to the work of Prat-García et al. [4]:

(1) $${\mathrm{ratio}}_{i}^{\mathrm{body}}=74.77\%\cdot \frac{w_{i}}{\sum_{n=1}^{N_{b}}w_{n}}$$

(2) $${\mathrm{ratio}}_{i}^{\mathrm{head}}=12.61\%\cdot \frac{2\cdot I_{i}}{0.28}$$

were w_n_ is the width of the n-th barrel body stave, N_b_ is the number of staves of the barrel body and 2·I_i_ is the total area of the i-th stave in m^2^.

### 3.2. Genetic Algorithms

Genetic algorithms (GAs) are iterative search heuristics that were proposed and developed by Holland and Goldberg based on the process of natural selection, and they are part of the larger class of evolutionary algorithms [27,28]. GAs are used to solve optimization problems by combining a randomized information exchange method with a survival-of-the-fittest strategy to identify optimal solutions of the considered problem.

The GA methods that are proposed in this article solve the problem of identifying the staves for the construction of barrel heads and bodies with a target global OTR. These methods implement the typically implemented genetic operators (crossover, mutation and selection), which are adapted to each problem. The individuals for both the body and head methods are arrays of variable length, where each array represents one head or body and where each element of the array has the index of one stave. In the next subsections, the remaining characteristics of the GA methods for constructing head and body barrels and the general implementation of the GA-based algorithms will be explained.

#### 3.2.1. Barrel Head Construction

The length of each solution in the barrel head construction GA method will have a variable odd number of stave indexes (typically between 7 and 11), which are in the same order as they are placed in the barrel head. The order of the staves of a head is important because it affects its global OTR and, in some cases, it could be impossible to construct the head because these staves in the specified order do not comply with the size requirements.

Three genetic operators were defined for the barrel heads: mutation, in which one stave of the head is replaced; external crossing, in which two heads are combined to generate a new head; and internal crossing, in which two staves of the head exchange their positions. A more detailed explanation of each genetic operator is presented in the next subsections.

##### Barrel Head Mutation

**Figure 1:**
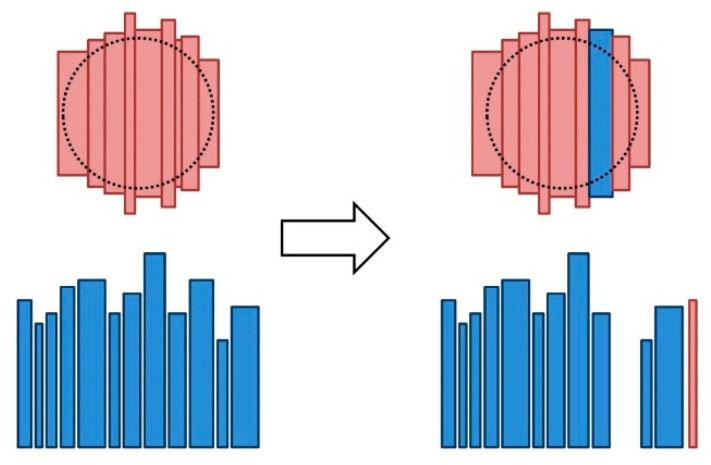
Procedure for applying the mutation operator to a head: one stave of the original head (represented in red) is randomly chosen, and it is replaced by a stave from the idle stave group (represented in blue) that complies the size requirements of the barrel head.

The barrel head mutation consists of the replacement of one stave of the head with a stave that has not been used in this head. This replacement must be conducted in consideration of the size requirements of a head barrel. Figure 1 illustrates this procedure.

To implement the barrel head mutation operator, the staves from the head were randomly ordered and, from the first stave to the last, the possibility of being replaced with each of the idle staves, which were also randomly ordered, was assessed; the execution of the mutation operator was complete when the replacement was possible. If the mutation was not possible after evaluating all the staves, the original head was regarded as the result of the mutation operator.

##### Barrel Head External Crossing

**Figure 2:**
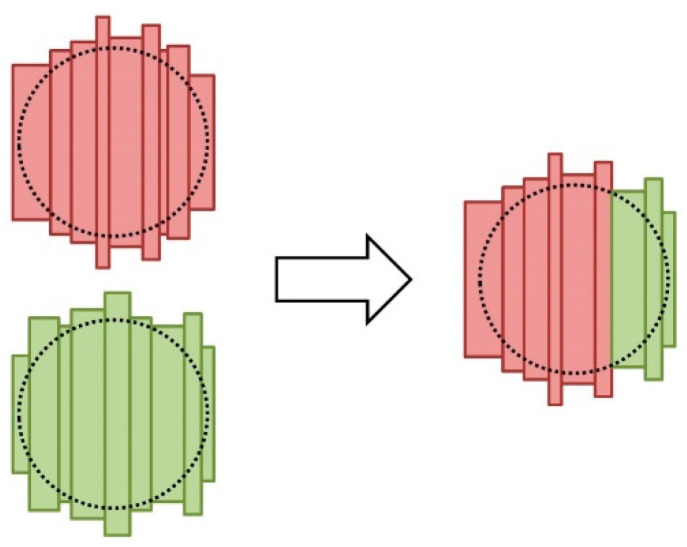
Procedure for applying the external crossing operator with two heads, with red and green staves. This procedure combines the staves of the two heads into a new head while attempting to maintain the positions of the staves and checking that the new distribution of the staves complies with the size requirements of the barrel head.

The barrel head external crossing consists of the combination of two barrel heads to build a third head. The resulting head, along with the barrel heads that were generated by the mutation operator, must comply the barrel head requirements. Moreover, as one or more staves could be present in the two heads to be combined, it must be checked in the resulting body that the same stave is not considered more than once. Figure 2 illustrates this procedure.

In the implemented barrel body external crossing operator, the stave position of the first barrel head is chosen as the limit of the staves of the first head that will be considered in the resulting head. Then, the staves of the second head are iteratively added to the resulting head while checking that they are not present in the resulting head because they were also present in the first stave. Finally, the remaining staves of the first head are also considered if they are needed to satisfy the size requirements when it is not possible to finish the head with the staves of the second head. If it is not possible to generate a new head with the external crossing operator, one of the two original heads is regarded as a result of this operator.

##### Barrel Head Internal Crossing

**Figure 3:**
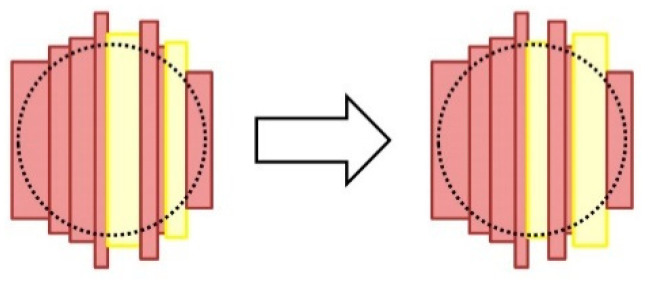
Procedure for applying the internal crossing operator to a head: two randomly chosen staves of the original head (represented in yellow) change their positions in the head if the new distribution of the staves complies the size requirements of the barrel head.

The barrel head internal crossing consists of changing the positions of two staves of a head when it is possible. Figure 3 illustrates this procedure.

In the implemented barrel body internal crossing operator, the two considered staves were randomly chosen, and whether the final distribution of the staves complies the requirements for the barrel head was checked. If the requirements are not satisfied after trying all the combinations of staves, the operator returns the original head as its result.

#### 3.2.2. Barrel Body Construction

The length of each solution in the barrel body construction GA method will correspond to the number of staves that satisfy the size requirements for the barrel body. The order of the staves for this element is irrelevant because it affects neither the global OTR nor its size requirements. Nevertheless, after applying the GA method to select the staves for the construction of a body, the selected staves could be reordered to distribute the staves with larger and smaller widths along the barrel body, as some cooperages used to do.

The initialization of a solution consists of finding a set of staves for which the total width is in the width range that is determined by the size requirements and there is at least one stave with sufficient width for placing the barrel bung. A width range of ±0.5 mm around the total width of 218 cm was considered in our experiments. Two genetic operators were defined for the barrel bodies: the mutation operator, which replaces several staves of the body, and the external crossing operator, which combines two bodies to generate a new body. In this case, the internal crossing operator was not considered because, unlike in the barrel heads, the order of staves in the barrel body does not affect its OTR.

##### Barrel Body Mutation

**Figure 4:**
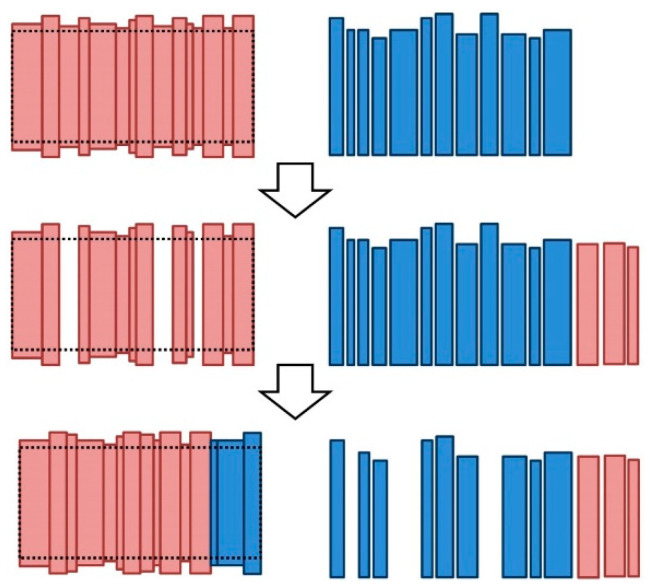
Procedure for applying the mutation operator to a body. First, several staves of the original body, which are represented in red, are randomly chosen and removed from the body. Then, a set of staves that are not used in this body, which are represented in blue, are chosen to replace the removed staves while checking that the total width of the new body is of the considered dimensions. The number of staves that are represented in this figure is lower than is typical in a real body (approximately 30) to improve the visibility.

The barrel body mutation consists of the replacement of several staves of a body with staves that have not been used in this body. This replacement must be conducted in consideration of the size requirements of the body barrel. Figure 4 illustrates this procedure.

With the application of the implemented barrel body mutation operator, three staves were always removed from a body. Moreover, to facilitate the implementation of the method, the first stave of the body, which is the stave with sufficient width for placement of the bung, was never replaced with the mutation operator. Hence, only the width requirement of the barrel body needed to be checked in this procedure.

##### Barrel Body External Crossing

**Figure 5:**
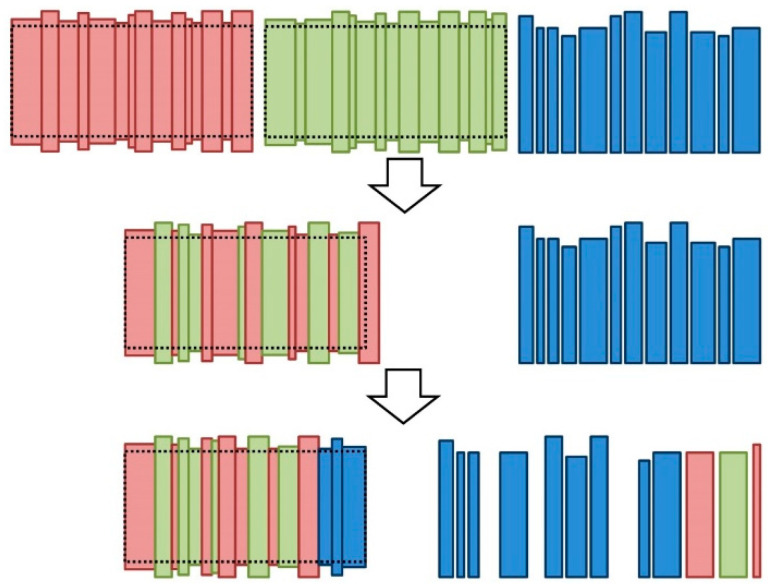
Procedure for applying the external crossing operator to two bodies, which are represented with red and green staves. First, staves for a new body are chosen from the staves of the two bodies randomly. Second, the width requirements of a body barrel are checked and, if these requirements are not satisfied, the mutation operator is applied to build a valid body using idle staves. The number of staves that are represented in this figure is lower than the typical number in a real body (approximately 30) to improve the visibility.

The barrel body external crossing consists of the combination of two barrel bodies to build a third barrel body. The resulting body, along with the barrel bodies that are generated with the mutation operator, must comply with the barrel body size requirements. Moreover, as one or more staves could be present in the two bodies to be combined, it must be checked in the resulting body that no stave is considered more than once. As it could be difficult to attain a valid width for the barrel body with a small set of staves, an additional mutation operator was considered to attempt to satisfy this requirement with extra staves. Figure 5 illustrates this procedure.

In the implemented barrel body external crossing operator, the first stave of one of the two bodies, which is the stave with sufficient width for placement of the bung, was randomly chosen as the first stave of the resulting body to satisfy the first size requirement of the body. Then, the remaining staves were considered, the duplicated staves were removed, and staves were randomly chosen for the construction of the new body until the minimum width was reached. If the final width exceeded the maximum allowed width, the mutation operator was applied to obtain a valid body by considering extra staves.

#### 3.2.3. GA-Based Method Implementation

Both the barrel head and barrel body construction methods were implemented according to the framework that will be described in this section.

The first part of the methods is the initialization of the individuals prior to the first iterative generation of solutions. Ten individuals were initialized in consideration of the idle staves and the size criteria. Then, the OTR of each head or body was calculated to obtain the fitness of each element applying the expression presented in Equation (3). The factor of −1 ensures that the quality of the solution increases with the fitness value.

(3) $$fitness=-\left|OTR_{target}-OTR_{head/body}\right|$$

The second part of the methods is the iteration of generations of the method. In every generation, the solution with the best fitness value, was always selected from the previous to the next generation. Then, the remaining 9 individuals for the next generation were generated by randomly using one of the following procedures: creating new solutions, reapplying a solution from the previous generation or applying genetic operators to the solutions of the previous generation. The probabilities of generating individuals for the next generation via the previously described procedures are listed in Table 1. These probabilities were chosen with a trial-and-error procedure, taking into account both the previous knowledge of the authors of the manuscript and the probabilities employed by other authors in the literature, in order to optimize the performance of the GA-based method as much as possible.

Moreover, some of these procedures must select one or more elements from the previous generation. This selection was conducted via the roulette wheel selection method, in which the probability of choosing an element is proportional to its fitness. In the proposed methods, the probability of choosing the n-th element was calculated according to Equation (4):

(4) $$\mathrm{probability}=\frac{e^{-\frac{f_{n}}{\min{\left\{f_{n}\right\}}_{n=1}^{N}}}}{\sum_{n=1}^{N}e^{-\frac{f_{n}}{\min{\left\{f_{n}\right\}}_{n=1}^{N}}}}$$

where f_n_ is the fitness of the n-th element and N the number of elements.

The third part of the method is the evaluation of the termination criteria. The iteration of the generations, which is conducted in the second part, was terminated in our experiments according to two termination criteria: iterating for longer than a specified iteration time or obtaining an OTR error that is smaller than a target error. The iteration time termination criterion was used in combination with the error criterion to avoid an infinite iteration loop.

**Figure 6:**
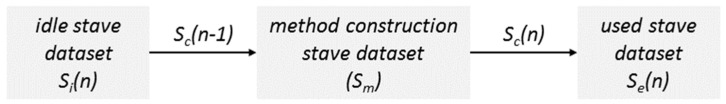
Stave datasets that are considered in the proposed construction methods.

Finally, the methods that are presented in this article construct one head or one body. Thus, they should be applied several times for the construction of several barrels. Each experiment will start by considering a set of *S_i_* staves, which will be referred to as the idle stave dataset. The initialization process consists of randomly choosing a set of *S_m_* staves from the idle stave dataset, which will be the staves that are used in the first iteration of the construction method and will be referred to as the construction method stave dataset. The number of staves of the construction method stave dataset (*S_m_*) will be constant during each construction process. After applying the construction method in the first iteration, the staves that are selected to build the body or the head, the number *S_c_(n)* of which can vary among iterations (*n*), will be moved to the used stave dataset and will not be used again in this experiment. Finally, after each iteration, the construction method stave dataset randomly chooses *S_c_(n)* staves from the idle stave dataset to replenish *S_m_* staves for the next iteration. The experiment iterates until a fixed number of elements have been constructed or until the number of staves in the idle stave dataset is zero. Figure 6 illustrates the three datasets and the number of staves in each dataset in each iteration. The idle stave dataset contains the staves that are available for use; the method construction stave dataset, which has a constant number *S_m_* of staves, contains the staves that are used by the method; and the used stave dataset contains the staves that have been used to construct barrel bodies or heads. For the idle stave dataset, the initial number of staves is *S_i_*(0) *= S_i_*, the number of staves in this dataset before the first iteration is *S_i_*(1) *= S_i_* − *S_m_*, and the number of staves before iteration *n* is the presented in Equation (5), while the number of staves in the used stave dataset before the first iteration is *S_e_*(1) = 0, and the number before iteration n is shown in Equation (6).

(5) $$S_{i}\left(n\right)=S_{i}-S_{m}-\sum_{k=2}^{n}S_{c}\left(k-1\right),\;n\geq 2$$

(6) $$S_{e}\left(n\right)=\sum_{k=2}^{n}S_{c}\left(n-1\right),\;n\geq 2$$

This simulation procedure simulates the normal working conditions of a production line, where there are constant input and output fluxes of elements on each link of the production chain.

### 3.3. GA-Based Method Comparison

The results that were obtained via the GA-based proposed methods were compared with the results that were obtained with other stave selection methods to evaluate their performances.

The first selection methods that were considered were the selection methods that are used in practice in cooperages. These methods, which will be referred to as *current*, consider only the lengths and the widths of the staves and do not consider their OTRs when looking for a set of staves that satisfy the barrel head or body size requirements.

The second selection methods that were considered were Monte Carlo selection methods, which will be referred to as MC in the next sections. Monte Carlo methods consider both the size and the OTR of each stave in the construction of a head or a body with a target OTR. They select random sets of staves and check if they satisfy all the proposed requirements. They were implemented by simplifying the proposed GA-based methods by considering only two individuals for each generation, generating a new solution with a probability of 100%, and reapplying solutions from the previous generation or applying mutation or crossing operators with a probability of 0%, according to Table 1. Via this approach, there are two individuals in every generation: the best from the previous generation and another randomly chosen individual. Hence, these methods consider random individuals and choose the best as the result.

## 4. Results and Discussion

Several scenarios were simulated to analyze the proposed stave selection method. These scenarios correspond to cases that could occur in a cooperage with the OTR of all the staves calculated or estimated. The next subsections will evaluate the performance of the proposed method in these scenarios.

### 4.1. Barrel Homogenization

One possible application of the proposed method is the homogenization of the OTR of the barrels that are constructed in a cooperage. Currently, the staves of the barrel are chosen randomly, which leads to a significant OTR variance among the resulting barrels.

**Figure 7:**
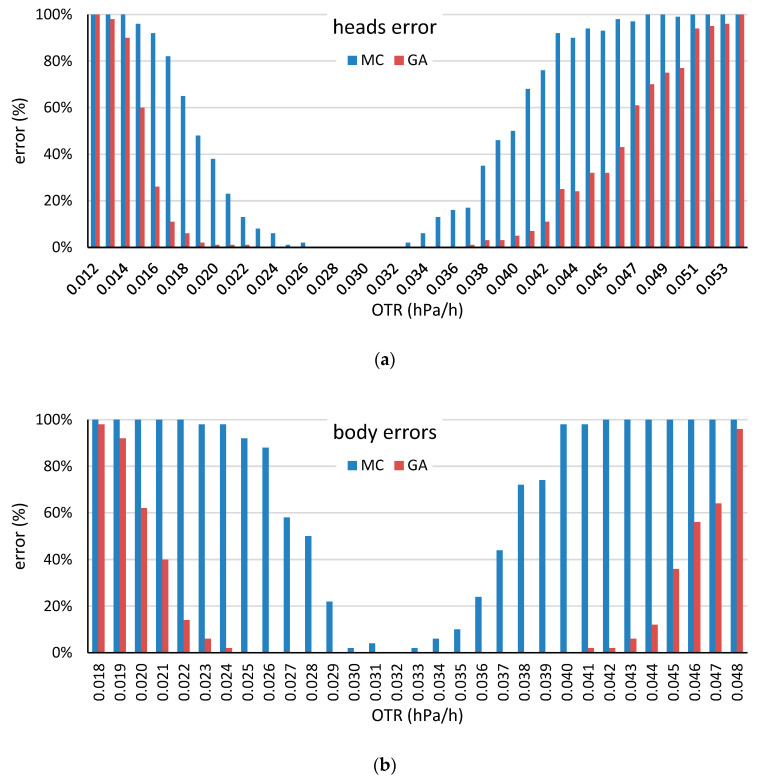
Error rates of the head stave (**a**) and the body stave (**b**) by selection method for the various target OTR values. A simulation was regarded as an error when the maximum iteration time was reached without identifying a head or a body barrel with the desired OTR value.

**Figure 8:**
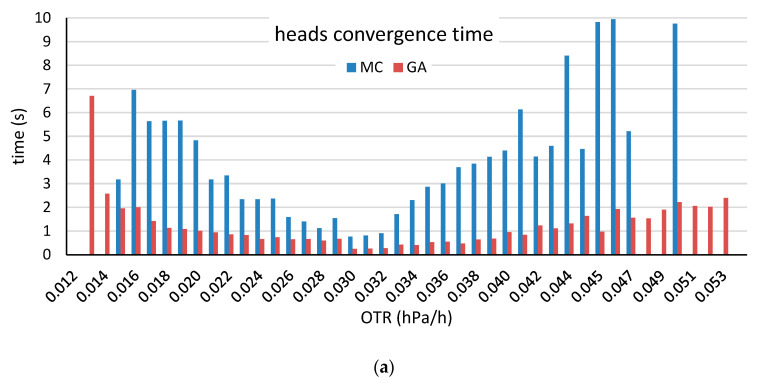

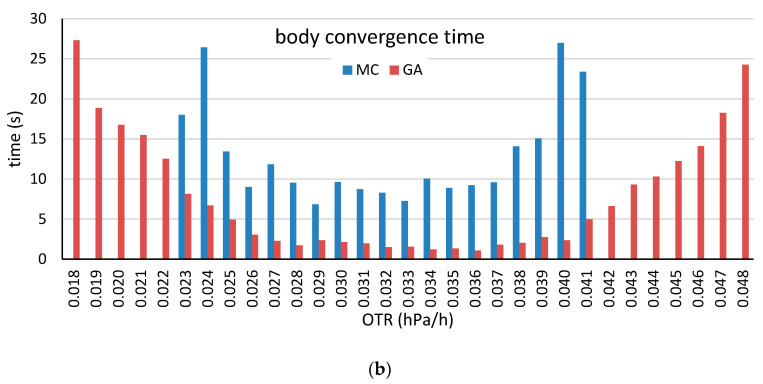
Mean convergence times of the head stave (**a**) and the body stave (**b**) by selection method for various target OTR values. The mean time was calculated considering only the heads and bodies that were constructed prior to reaching the maximum iteration time.

Thus, in this first scenario, the objective of the stave selection method will be the creation of heads and bodies with OTRs that are similar to the mean OTRs of all the head and body staves, respectively. The proposed GA-based selection methods were simulated and compared with the *current* and the MC-based selection methods. The target OTR values for the MC-based and the GA-based methods were chosen as the mean OTR values for the body and the head staves, which are 0.03275 and 0.02928 hPa/h, respectively, as calculated in [4]. The construction of 50 barrels (50 body staves and 100 body heads) was simulated with various numbers of staves and various processing times in the selection method. Table 2 and Table 3 present the obtained results for the heads and bodies construction simulation respectively. Moreover, Figure 7 and Figure 8 show the performance of both MC-based and GA-based methods when the target OTR varies.

Analyzing the results in Table 2 and Table 3, several observations are made. Analyzing the mean value of the bodies and heads, these values are close to the target OTR in the three methods. This finding could be explained by the central limit theorem that denotes that the mean value of the barrels and bodies will be close to the mean value of the staves even for the *current* method, in which the OTR is not considered in the construction of the barrel element. However, analyzing the coefficient of variation there are differences among the methods considered. First, there are large differences between the coefficient of variation for the *current* method and the coefficients of variation for the MC and GA methods, which is expected because the latter two methods consider the OTR of the staves in the barrel element construction. Moreover, this coefficient of variation, which is between 14% and 17% for the heads and between 6% and 9% for the bodies, is an approximation of the actual variance of the barrels that are constructed in a cooperage. The second observation in comparing the methods is that, despite the small coefficients of variations for the MC and GA methods, there is a consistent difference between them: the coefficient of variation for the GA-based methods is always smaller. Furthermore, the GA-based compared with the MC-based methods performance is better when the absolute difference between the OTR and the mean OTR increases, as it can be seen in Figure 7 and Figure 8. This observation shows the utility and robustness of the proposed GA-based methods.

Comparing the simulation parameters that are considered for the MC and GA methods, increasing the simulation time reduces the variability of the constructed elements because the construction methods have more time to perform iterations and the probability of finding better solutions increases. Nevertheless, increasing the number of iterations increases the processing time of the method, which is an undesirable feature of the method. Moreover, analyzing the number of staves that are considered in the method, the variability is reduced as the number of staves increases because it is easier to find staves with satisfactory characteristics for the construction of an element, but the increment of the staves makes the process more complex to implement in a cooperage due to the storage and the logistical requirements that are associated with this modification. For these reasons, choosing the iteration time and the number of staves is a compromise solution. In the next experiments of this article, we are going to use an iteration time of 1 s, 50 staves for the head stave selection method, and 100 staves for the body stave selection method.

Finally, by comparing the construction of the heads and bodies, interesting differences are identified. The coefficient of variation for the heads is larger than the coefficient of variation for the bodies for the *current* method. This finding could be explained by the bodies having approximately 3 times more staves than the heads; hence, the variance when a set of staves is randomly chosen decreases when the number of staves increases. Nevertheless, it is difficult to draw a similar conclusion when comparing the MC-based and the GA-based methods. In these cases, there are differences among the stave selection criteria that are considered that render impossible the comparison of the head and the body construction methods in the same conditions to analyze the differences of the obtained coefficient of variation values.

### 4.2. Low-OTR and High-OTR Barrel Production

The second scenario was proposed to construct low-OTR (L-OTR) and high-OTR (H-OTR) barrels in the regular production of a cooperage. The body and the head staves were preclassified into three groups of staves each: low-OTR, mid-OTR, and high-OTR body and head staves, respectively, repeating the strategy of Prat-García et al. [4] by using the OTR estimation method proposed in [19]. In our work, the threshold values that were considered in the formation of the groups were 0.0157 and 0.0396 hPa/h for the body staves and 0.0230 and 0.0434 hPa/h for the head staves, which were the same threshold values utilized in the work of [4].

**Figure 9:**
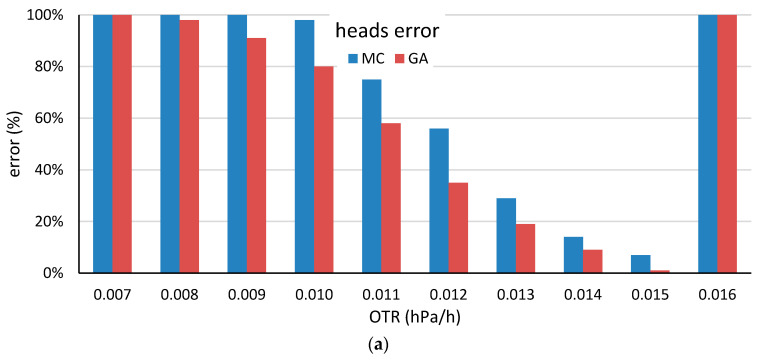

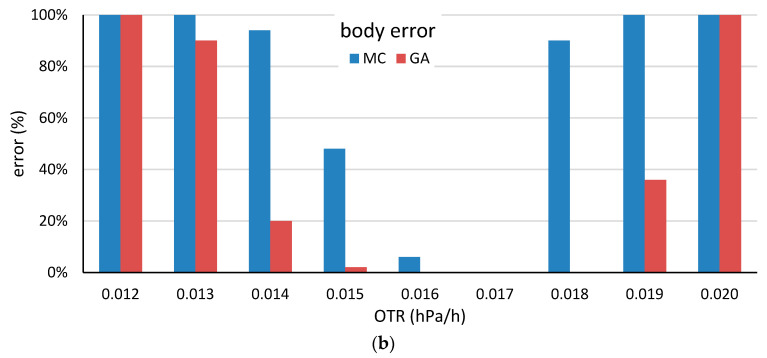
Error rates of the for the head stave (**a**) and the body stave (**b**) by selection method for various target OTR values obtained by using the head and body low-OTR staves. A simulation was regarded as an error if the maximum iteration time was reached without identifying a head or a body barrel with the desired OTR value.

**Figure 10:**
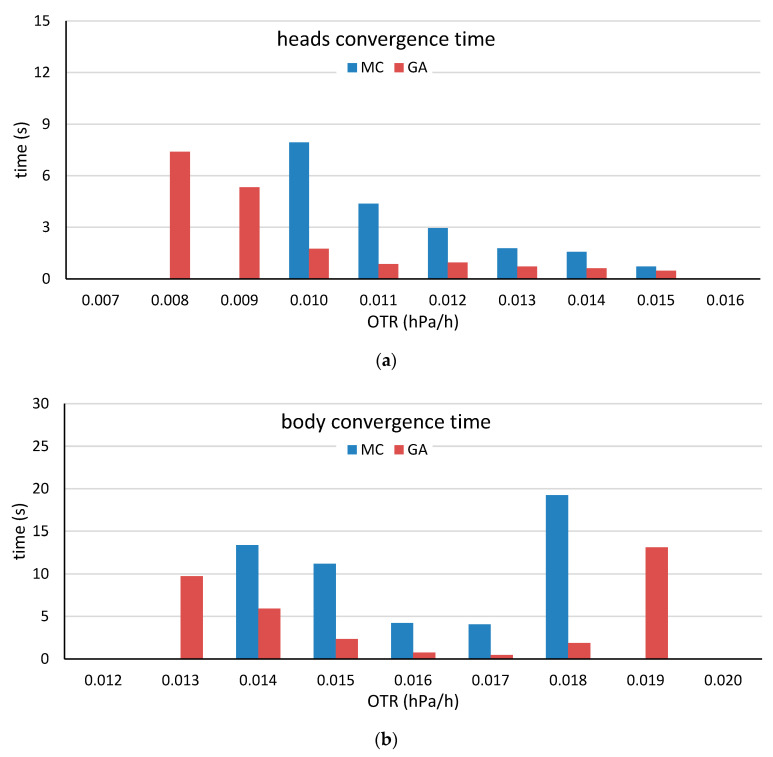
Mean convergence times of the for the head stave (**a**) and the body stave (**b**) by selection method for various target OTR values obtained by using the head and body low-OTR staves. The mean time was calculated with consideration of only the heads and bodies that were constructed before reaching the maximum iteration time.

**Figure 11:**
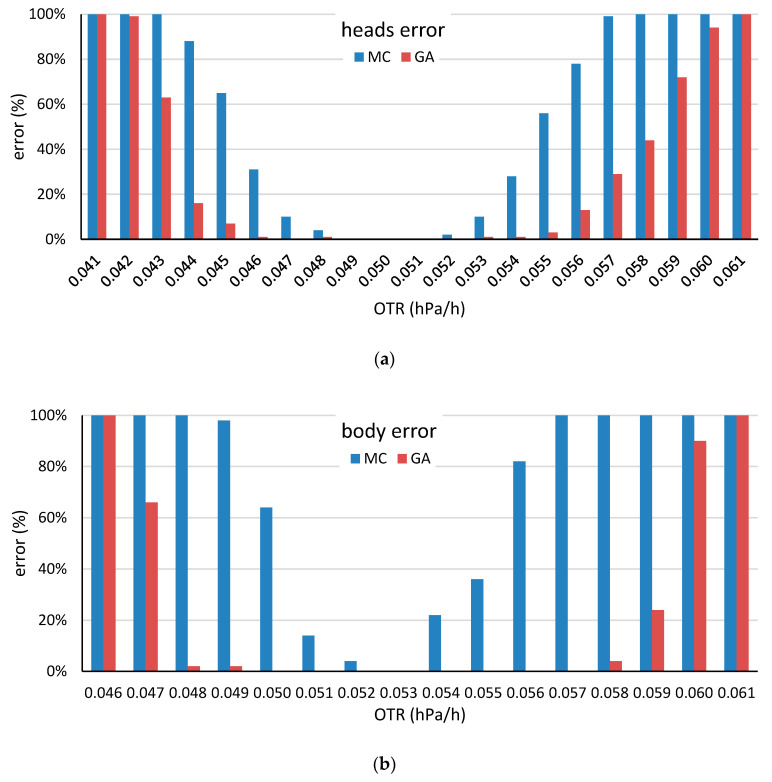
Error rates of the for the head stave (**a**) and the body stave (**b**) by selection method for various target OTR values obtained by using the head and body high-OTR staves. A simulation was regarded as an error if the maximum iteration time was reached without identifying a head or a body barrel with the desired OTR value.

**Figure 12:**
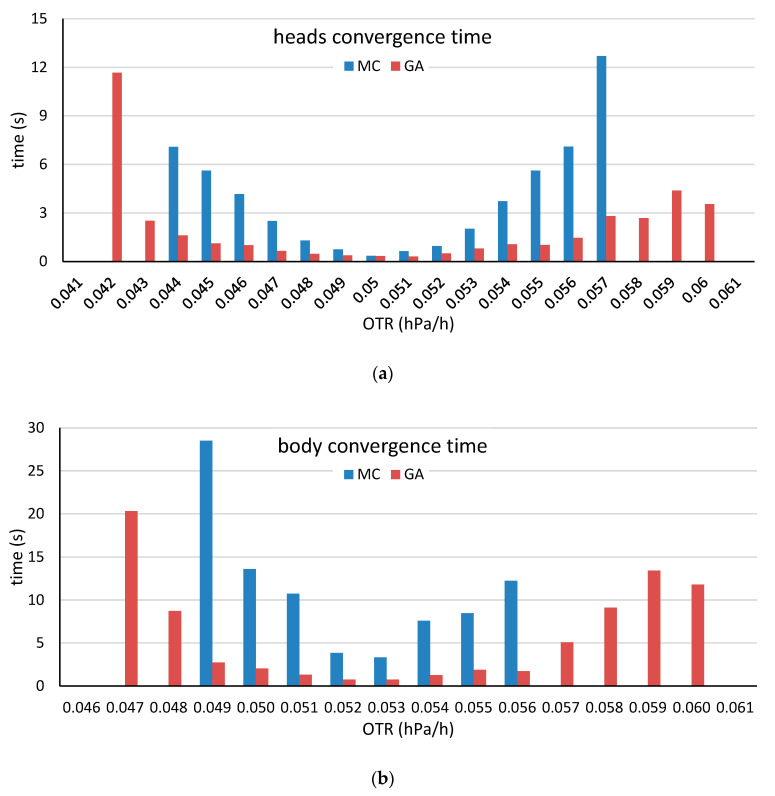
Mean convergence times of the head stave (**a**) and the body stave (**b**) by selection method for various target OTR values obtained by using the head and body high-OTR staves. The mean time was calculated in consideration of only the heads and bodies that were constructed before reaching the maximum iteration time.

After preclassifying the staves, the proposed methods were applied to homogenize the OTR of the constructed barrels in a similar way to the procedure followed in the first scenario. The previously selected parameters of 25 and 50 staves for the head and body construction methods, respectively, were chosen, and a maximum iteration time of 1 s was set in both cases. Moreover, the target OTR value was chosen as the mean OTR value of the staves that were considered in each case, which was 0.0148985 and 0.0165681 hPa/h for the L-OTR head and body, respectively, and 0.0501335 and 0.0528710 hPa/h for the H-OTR head and body, respectively. Table 4 presents the obtained results for the L-OTR and the H-OTR barrels, while Figure 9, Figure 10, Figure 11 and Figure 12 show the performance of MC-based and GA based methods when the target OTR varies.

First, analyzing the obtained results and comparing them with the results in Section 4.1, several observations from the previous subsection are also valid for this experiment: the evaluated MC and GA methods significantly reduce the variability of the constructed elements according to a comparison of their coefficients of variation with those obtained by the *current* method. Moreover, the proposed GA methods improve the performance of the MC methods, being also more robust to changes in the target OTR.

Second, the obtained results demonstrate the advantages of preclassifying staves as L-OTR and H-OTR. Comparing the obtained results with those presented in Figure 7 and Figure 8, preclassifying staves is the best strategy if the construction of barrels with two OTR levels is the objective. The first reason to justify it is the high error rate obtained in Figure 7 and Figure 8 for the extreme OTR values. The second reason is that it is possible to construct L-OTR and H-OTR barrels via the *current* selection method. Nevertheless, the difference in the variability that is presented in Table 4 suggests that the proposed GA-based method should be implemented.

Third, the L-OTR and the H-OTR barrels differ as follows: the coefficients of variation are smaller for the H-OTR heads and bodies. This is mainly due to the difference in the mean OTR values, which are between 3 and 4 times greater for the H-OTR elements; hence, the variability, which is regarded as the standard deviation, is larger for the H-OTR barrel elements.

### 4.3. Real Cooperage Simulation

The last scenario that was considered in the evaluation of the proposed method was the production of 50 wine barrels, which is a regular daily production of cooperages such as INTONA S.L. cooperage. Fifty L-OTR and 50 H-OTR barrels with the same characteristics as in experiment 4.2 were constructed: 25 and 50 staves were regarded as the method dataset for the head and the body construction methods, with a maximum iteration time of 1 s and target OTR values of 0.0148985 and 0.0165681 hPa/h for the L-OTR head and body, respectively, and 0.0501335 and 0.0528710 hPa/h for the H-OTR head and body, respectively. These configuration parameters are the same as those that were chosen in the previous scenarios.

The method procedure was similar to the procedures for the previous scenarios but with the generation of 100 heads or 50 bodies per simulation instead of one. 2000 L-OTR and 2000 H-OTR stave samples for both the head and the body were considered, which were generated based on the CDF of the features of the original data according to the procedure that is described in Section 2.1. These samples were organized as presented in Figure 6; hence, at the beginning of the experiment, all the staves were in the idle stave dataset. From this dataset, 25 or 50 staves for the heads or the bodies, respectively, were randomly extracted to the method construction stave dataset for the construction of a head or a body. After the stave selection methods had been executed, the selected staves were moved to the used stave dataset, and the remaining staves were moved to the idle stave dataset. This procedure was iteratively repeated until all the heads and bodies were constructed, and it was applied for both the MC-based methods and the GA-based methods.

The 2000 samples of each experiment were randomly ordered at the beginning of the experiment in an ordered queue. With this randomly initialized queue for the idle stave dataset, the method construction stave dataset always selected the first 25 or 50 staves of this queue and moved the unused staves after the application of the method to the last position of the idle stave dataset queue. This procedure was applied to the MC-based methods and the GA-based methods with the same initial idle stave dataset queue to obtain similar conditions and to avoid differences in the comparison that are due to differences in the orders of the dataset. Moreover, the proposed queue simulates the production line of a cooperage; hence, it could be implemented on the production line.

Table 5 presents the results that were obtained in this experiment. These results provide an example of the performance that could be realized by the proposed methods in a real cooperage.

The first observation is that the GA-based methods outperform the MC-based methods in the real simulation.

The second observation regards the homogeneity of the obtained barrels. On the one hand, it can be seen that the coefficient of variation is very low, which is an important parameter for the homogeneity. On the other hand, when analyzing the worst cases, the homogeneity is again highly satisfactory: the variation range between the minimum and the maximum OTR values of the barrels is less than 1.3% with respect to the mean OTR.

The last observation regards the head and body assembly procedure. In this scenario, each barrel was constructed with the first available body and heads, e.g., the first barrel with the first body and the first and second heads and the second barrel with the second body and the third and fourth heads. It will be possible to reduce the variability of the resulting barrels by selecting the heads and bodies that are used to build each barrel to compensate the OTR values of the elements of the barrels. Nevertheless, implementing this procedure in a real cooperage will be difficult because it will be necessary to store several heads and bodies and to manage them for its assembly; it may not be worth applying this approach because sufficiently low variability can be realized without using this method.

## 5. Conclusions

The results that are presented in this article demonstrate that artificial intelligence methods that were implemented by using the genetic algorithm could be employed in real production to select the staves that are needed for the construction of barrels with various OTR values. The proposed methods can select the staves for the construction of the heads and the body of a barrel with one second of processing time for each element. Moreover, the experimental results demonstrate the performance of the proposed methods in constructing barrels with a desired OTR value, which significantly outperform brute-force MC methods. Finally, the preclassification of the staves according to their OTR values enabled the construction of low-OTR and high-OTR barrels, with mean OTR values of 0.016 and 0.052 hPa/h, respectively, and coefficients of variation of 0.10% and 0.18%, respectively.

## Figures and Tables

**Table 1:** Probabilities of using the five procedures to generate an individual for the next generation for the head and the body construction methods.

Procedure	Head	Body
Generating a new solution	40.0%	40.0%
Reapplying a solution from the previous generation	12.0%	13.4%
Applying the mutation operator	21.0%	23.3%
Applying the internal crossing operator	6.0%	0.0%
Applying the external crossing operator	21.0%	23.3%

**Table 2:** Results of the construction of 100 heads with a target OTR of 0.02928 hPa/h. The parameter “method staves” refers to the number of staves that are considered in the method dataset (Figure 6), and the time refers to the number of seconds that the methods iterate prior to outputting the best solution that has been found during this time.

Method Staves	Time (s)	Mean			Coefficient of Variation		
		*Current*	MC	GA	*Current*	MC	GA
25	1	0.029107	0.029219	0.029252	15.53%	1.4264%	0.8387%
50	1	0.028177	0.029283	0.029282	14.83%	0.0948%	0.0618%
150	1	0.029049	0.029279	0.029280	15.86%	0.0443%	0.0131%
25	5	0.028636	0.029268	0.029270	15.72%	0.4678%	0.3351%
50	5	0.028052	0.029279	0.029280	15.64%	0.0275%	0.0030%
150	5	0.028852	0.029280	0.029280	16.79%	0.0079%	0.0020%

**Table 3:** Results of the construction of 50 bodies with a target OTR of 0.03275 hPa/h. The parameter staves method refers to the number of staves that are considered in the method dataset (Figure 6), and the time refers to the number of seconds that the methods iterate prior to outputting the best solution that has been found during this time.

Method Staves	Time (s)	Mean			Coefficient of Variation		
		*Current*	MC	GA	*Current*	MC	GA
**50**	1	0.032259	0.032711	0.032789	7.749%	1.7901%	1.0290%
100	1	0.032094	0.032744	0.032734	6.716%	0.8282%	0.1383%
300	1	0.032538	0.032737	0.032746	7.752%	0.4966%	0.1090%
50	5	0.032762	0.032776	0.032751	8.053%	0.4985%	0.0218%
100	5	0.032650	0.032743	0.032749	7.289%	0.1223%	0.0263%
300	5	0.032412	0.032757	0.032750	6.751%	0.1030%	0.0166%

**Table 4:** Results of the construction of the heads and bodies for 50 low-OTR (L-OTR) or high-OTR (H-OTR) barrels.

Barrel Element	Stave Type	Target OTR	Mean			Coefficient of Variation		
			*Current*	MC	GA	*Current*	MC	GA
Heads	L-OTR	0.0148985	0.014750	0.014917	0.014915	5.585%	0.6261%	0.4849%
Body	L-OTR	0.0165681	0.016483	0.016583	0.016566	3.155%	0.2608%	0.0650%
Heads	H-OTR	0.0501335	0.050293	0.050133	0.050133	4.178%	0.0298%	0.0284%
Body	H-OTR	0.0528710	0.052992	0.052873	0.052869	1.804%	0.1110%	0.0387%

**Table 5:** Results of the real cooperage simulation, where the OTRs of 50 barrels that were constructed via the proposed methods were analyzed with their mean values, standard deviations, coefficients of variation, and minimum and maximum values.

	L-OTR		H-OTR	
	MC	GA	MC	GA
mean	0.01615057	0.01614661	0.05216888	0.05219895
standard deviation	2.6795 × 10^−5^	1.6284 × 10^−5^	1.0365 × 10^−4^	9.1959 × 10^−5^
coefficient of variation	0.16591%	0.10085%	0.19868%	0.17617%
minimum	0.01607707	0.01608685	0.05182518	0.05179106
maximum	0.01626386	0.01622588	0.05243214	0.05244636
